# Louisville Summer School of Medicine

**Published:** 1845-07

**Authors:** 


### LOUISVILLE SUMMER SCHOOL OF MEDICINE.

The Spring term of the Louisville Summer School of Medicine has just closed, and the Fall term will commence on the first Monday in September. A number of Students have arrived in the city in the last month, and many more will come in before the opening of the course in the autumn. The popularity of the Summer school is increasing; the class this season has been larger than that of the former year, and it is evident that, with due diligence on the part of those engaged in it, this may be rendered a most important source of instruction to students, especially in clinical medicine. The growing population of Louisville, its increasing commerce, and the consequent multiplication of Hospital patients, are circumstances which must give it an annually increased claim upon the attention of students desirous of knowledge in the practical branches of their profession. Y.

